# Designing socially assistive robots for clinical practice: insights from an asynchronous remote community of speech-language pathologists

**DOI:** 10.3389/frobt.2025.1646880

**Published:** 2025-10-01

**Authors:** Denielle Oliva, Abbie Olszewski, Shekoufeh Sadeghi, Karthik Dantu, David Feil-Seifer

**Affiliations:** 1 Socially Assistive Robotics Group, University of Nevada, Computer Science and Engineering, Reno, NV, United States; 2 Literacy Studies, College of Education and Human Development, University of Nevada, Reno, NV, United States; 3 Drones Lab, Computer Science and Engineering, University of Buffalo, Buffalo, NY, United States

**Keywords:** socially assistive robots, speech-language pathology, co-design, expression, asynchronous remote community

## Abstract

**Introduction:**

Socially Assistive Robots (SARs) hold promise for augmenting speech-language therapy by addressing high caseloads and enhancing child engagement. However, many implementations remain misaligned with clinician practices and overlook expressive strategies central to speech-language pathology.

**Methods:**

We conducted a 4-week Asynchronous Remote Community (ARC) study with thirteen licensed speech-language pathologists (SLPs). Participants engaged in weekly activities and asynchronous discussions, contributing reflective insights on emotional expression, domain-specific needs, and potential roles for SARs. The ARC format supported distributed, flexible engagement and facilitated iterative co-design through longitudinal peer dialogue. Data were analyzed using thematic analysis to identify emerging patterns.

**Results:**

Analysis revealed five clinician-driven design considerations for SARs: (1) the need for expressive and multi-modal communication; (2) customization of behaviors to accommodate sensory and developmental profiles; (3) adaptability of roles across therapy contexts; (4) ethical concerns surrounding overuse and fears of clinician replacement; and (5) opportunities for data tracking and personalization.

**Discussion:**

Findings highlight clinician-informed design implications that can guide the development of socially intelligent, adaptable, and ethically grounded SARs. The ARC approach proved a viable co-design framework, enabling deeper reflection and peer-driven requirements than traditional short-term methods. This work bridges the gap between robotic capabilities and clinical expectations, underscoring the importance of embedding clinician expertise in SAR design to foster meaningful integration into speech-language interventions.

## 1 Introduction

Speech-language pathologists (SLPs) are confronted with mounting difficulties in addressing the diverse and increasing demands of their caseloads^1^. Both in clinics and educational settings, SLPs commonly report large student-to-therapist ratios, insufficient resources, and time limitations that curtail their provision of individualized, high-quality services^2^. The shortage of licensed SLPs has compounded these problems, especially in rural or underserved areas (Squires, 2013). This limited amount of bandwidth not only restricts access to services but also places colossal strain on practicing clinicians, who have to manage enormous documentation demands, diverse client needs, and administrative tasks in addition to direct intervention.

These workforce constraints underscore the demand for innovative support tools that can expand therapist reach without compromise to therapeutic integrity. Among these tools, socially assistive robots (SARs) represent a promising strategy for augmenting therapeutic delivery (Feil-Seifer and Matarić, 2005).

SARs are designed to socially engage with the user, offering verbal prompts, modeling behavior, and encouragement through embodied, interactive communication (Feil-Seifer and Matarić, 2005). Compared to tablet apps or static visual displays, SARs offer real-time responsiveness and bodily presence, enabling novel forms of engagement and scaffolding in therapy settings (Wainer et al., 2006; 2007). Their potential lies in their ability to support the therapist as a consistent, motivating, and adaptive partner.

Despite their promise, SARs remain rare in the majority of speech-language pathology clinics. One key obstacle is the lack of substantial input of clinician experience into the design and use of these technologies (Šabanović et al., 2014; Jeong et al., 2017). In most instances, SAR development concentrates on engineering requirements more than the multifaceted demands of clinical practice (Scassellati et al., 2012). This has led to a discrepancy between what SARs can do and what therapists need. SLPs work in very dynamic, relational arenas where moment-to-moment changes in tone, affect, and pacing are responsive to client progress (Greenwell and Walsh, 2021). Tools that do not recognize or respond to this level of complexity may be unsuitable for adoption or unable to offer much support.

One of the underlying reasons for this disparity lies in the inherently interpersonal nature of speech-language therapy. SLPs rely intensely on social signals, affective responsiveness, and fine-grained facial expressions to guide therapy (Friedman et al., 1980; Buck et al., 1972). Such interactions are at the heart of establishing trust, scaffolding communication, and responding dynamically to each child’s requirements. Facial expression, emotional tone, and interactive timing are not peripheral characteristics; they are central to therapeutic effectiveness, specially in SAR assisted therapy intervention (Saerbeck et al., 2010; Kennedy et al., 2017; Kalegina et al., 2018). Children especially show a sensitivity to affective cues, imitating emotional tone and nonverbal expressions as they learn to regulate their own communication and behavior (Camras, 1985; Chris, 2009). These, while necessary, are not solved problems in robotics.

With these stakes in mind, it is imperative that SAR development utilize a clinician-centered design process grounded in the daily experiences and clinical wisdom of SLPs. Clinicians bring a nuanced understanding of therapeutic pacing, emotional rapport, and intervention goals that should inform both the behavior and aesthetics of SARs. Yet, the majority of existing applications focus narrowly on ASD populations or isolated speech activities, often without integration into broader therapy goals. Many are evaluated primarily on short-term engagement metrics rather than long-term clinical outcomes or alignment with therapist workflows (Getson and Nejat, 2022). This disconnect underscores the need for an SLP-led, iterative design model that develops robot capabilities to preserve therapeutic goals, sustain engagement, and respect clinical processes (Spitale et al., 2023).

Such a model would integrate SARs into therapeutic models, documentation protocols, and the dynamic interpersonal styles employed by SLPs, not as agents of novelty or automation but as co-facilitators. Designed in this manner, SARs have the potential to enhance the therapist’s capacity to model behavior, regulate affect, and scaffold practice during session. Their non-threatening embodiments, predictable behavior, and consistent prompt delivery make them particularly well-suited for repetitive skill-practice exercises (Scassellati et al., 2012). At the heart of this work is an appreciation for how SARs might harmonize with the natural rhythms of therapy—augmenting session objectives, delivering behavioral prompts, and reinforcing scaffolding initiated by clinicians.

This study analyzes clinicians’ attitudes, needs, and priorities for SAR use in speech-language therapy through responses collected in an ARC study. Through thematic analysis of multi-week discussion responses, we aim to reveal the priorities, concerns, and environmental considerations that should guide the next-generation of socially assistive technologies for therapy. These results offer a critical foundation for human-robot interaction (HRI) design for real-world practice in speech-language therapy.

This study employed a mixed-methods design to investigate how practicing SLPs perceive the potential roles and design features of SARs in therapeutic contexts. To gather rich, practitioner-informed data while accommodating clinical workloads, we utilized an ARC model over a 4-week period.

We aimed to explore the following research questions.

• RQ1: What experiences from an SLP’s typical routine can inform the design of a SAR?
• RQ2: How does an SLP encourage engagement through nonverbal communication?
• RQ3: How do the experiences of SLPs influence their opinions and willingness to use a SAR during interventions?
• RQ4: What is required for the acceptance and usability of a SAR agent during an intervention?
• RQ5: What design considerations would be most helpful based on the preferences of SLPs?

## 2 Background

### 2.1 Socially assistive robotics in pediatric therapy

Socially assistive robots (SARs) have been a novel approach to improving interaction and therapy results in children’s therapy environments. Previous research has demonstrated the potential of SARs in helping children with autism spectrum disorder (ASD) learn social communication skills, stay engaged, and adhere to organized routines. These robots, often shaped in the form of welcoming embodiments and scripted cues, provide a consistent and non-judgmental interaction partner, thus extremely useful for repetitive skill-building exercises (Shi et al., 2022). Research has also explored SAR inclusion in physical rehabilitation and educational environments, showing how embodied interaction can facilitate engagement (Riches et al., 2021). However, the majority of SAR applications in pediatric treatment are limited in scope, usually created and piloted in very controlled environments with little clinician involvement (Clabaugh and Matarić, 2019).

Some examples of SAR platforms utilized in therapy are robots such as Kaspar, NAO, and Moxie, which have demonstrated encouraging outcomes in interacting with children using predefined turn-taking, imitation, and role-playing (Cano et al., 2021).

SARs have also showed to integrate well into physical rehabilitation and education, demonstrating the way embodied interaction can engage people to take part (Langer et al., 2019). Still, the majority of SAR applications for pediatric therapy are narrow in scope, tested only in highly controlled situations with minimal clinician involvement in development (Rudovic et al., 2018). This work aims to extend the SAR paradigm by embedding design in the actual-world constraints and values of speech-language pathology.

### 2.2 Nonverbal communication in SLP

Nonverbal communication is a foundational piece of effective speech-language pathology, particularly in pediatric settings where children will start to utilize gestures, facial expressions, and prosody as means of communication before verbal fluency (Iverson and Goldin-Meadow, 2005; Clough and Duff, 2020). Nonverbal communication is utilized by SLPs to model behavior, to convey emotional tone, and to facilitate social interaction skill scaffolding (Paul, 2007; Rowe and Goldin-Meadow, 2012). Earlier research has noted how children will mirror clinician expressions and posture during early communication tasks, substantiating the importance of affective alignment and visual attention (Feldman, 2007; Meltzoff and Decety, 2003).

In child therapy, effective engagement is often a question of timing, tone, and synchrony—subtle nuances that determine whether a prompt is felt as supportive or intrusive. SLPs often modify facial expressions and prosodic variation moment by moment as a function of child affect or attention state. These modifications work to stabilize a child’s regulatory balance and optimize therapeutic gains (Beukelman and Mirenda, 2012; Ammaniti and Ferrari, 2013). The emerging discipline of affective computing is seeking to reinstate these cues into robotic systems, however, with a notable distinction in achieving emotional fluidity and expressiveness characteristic of a trained clinician assistant (Breazeal, 2003; Picard, 1997).

SARs will therefore need to be able to detect, interpret, and produce nonverbal cues in a way that is not just correct but contextually suitable. For instance, a tardy or exaggerated smile may perplex the child and fail to reinforce the behavior. Furthermore, SLPs deftly utilize silence, gaze, and body proximity to regulate turn-taking or encourage self-management. Replicating or supplementing such practices with SARs will require strong sensors, refined timing of output, and therapist adaptation capabilities.

This work expands on this understanding by exploring how therapists envision using SARs as supplements to—but not replacements for—nonverbal engagement strategies in therapy. By basing SAR interaction design on the unobtrusive choreography of therapeutic nonverbal communication, designers can create systems that are emotionally congruent, socially legible, and therapeutically effective. SLPs utilize nonverbal behaviors to model action, convey emotional tone, and scaffold social interaction skills (Norbury, 2014). Previous studies have also highlighted children’s mimicry of clinician affect and body position during early communication activities, which further contributes to the importance of affective concordance and eye gaze (Feldman, 2007). This interactive phenomenon poses a unique challenge to SAR development: robot systems must not only understand but also credibly express human-like affective cues in order to be accepted as therapeutic agents (Paiva et al., 2017). Human-robot interaction experiments suggest that children are sensitive to robot expressiveness incongruence, particularly when timing, gaze, or facial expressions are not natural (Kennedy et al., 2017).

### 2.3 Co-design in healthcare robotics

The co-design of robotic systems specifically developed for healthcare settings has been of particular interest as academics and professionals seek to bridge technological innovation with real-world applicability (MacLeod et al., 2016) Co-design emphasizes the value of incorporating end users—namely, healthcare professionals—into a recurring development process (Gasteiger et al., 2022). In healthcare robotics, co-design has proven effective in enriching devices’ relevance, building users’ sense of ownership, and disclosing latent limitations that may affect the implementation process.

Recent contributions (Lukkien et al., 2024) report on the importance of inclusive, context sensitive co-design methods, specifically when working with vulnerable populations like people with dementia. Their work stresses the need for responsive, ethically sensitive data gathering, and iterative design processes. Similarly, the responsible innovation framework (Stilgoe et al., 2013) draws on principles of anticipation, reflexivity, inclusion, and responsiveness. This co-design framework used in previous work (Suijkerbuijk et al., 2024) can be relevant for long-term care environments where ethical concerns, user autonomy, and societal values need to be balanced.

In rehabilitation and elder care applications, for example, co-design has yielded important robot embodiment, prompting systems, and session alignment improvements (Vandemeulebroucke et al., 2018). These processes generally include workshops, usability testing, and pilot deployments in which clinicians provide iterative feedback on how well robotic systems meet clinical requirements (Dalsgaard et al., 2022). By engaging users as co-creators rather than passive recipients, these strategies build systems that more effectively capture the subtleties of practice.

Despite greater recognition of its usefulness, co-design is underutilized in pediatric speech-language intervention to create SAR, partly due to the fact that much robotic design still takes place in laboratory settings with little clinician input until relatively late in the process (Suh et al., 2024). This can result in sophisticated technology but poorly matched equipment to therapeutic rhythms, constraints, and deeper ethical alignment. This study addresses this need by placing longitudinal co-design in the context of SLPs’ work across weeks of reflection and ideation. The resulting themes identify some of the affordances, dangers, and workflow concerns that may not be evident with short-term trials or lab-based observation alone.

Lastly, co-design not only assures more usability but deeper ethical alignment. Engaging therapists guarantees SARs operate within professional values, maintaining child dignity, and reducing undesired effects. As SAR competencies expand, increasingly, it will be important to anchor design in clinician insight—not just for adoption efficacy, but to protect the relational heart of therapeutic care. Co-design emphasizes end-user participatory involvement—here, clinicians—throughout the iterative development process (Rodriguez et al., 2023). Within healthcare robotics, co-design has been shown to increase device applicability, facilitate user ownership, and reveal unexpected constraints that may impede implementation (Gasteiger et al., 2022). In rehabilitation and geriatric healthcare, co-design sessions have influenced robot embodiment details, communication methods, and session protocols (Olatunji et al., 2024). Despite these advances, comparatively few SAR projects for speech therapy have adopted a co-design methodology. Our study addresses this gap by using multi-week engagement with practicing SLPs to uncover nuanced needs, guide feature development, and explore possible adoption barriers early in the design process.

## 3 Methods

This work investigates how SLPs foresee the design, usage, and integration of SARs into therapy through a 4 week ARC with practicing clinicians. This was developed as a co-design ARC approach, which allowed participants to reflect on prompts and surveys focused on concepts intended to draw out varied input. Our methods were informed by thematic analysis and sought to elicit both explicit needs and latent expectations of SLPs who work with varying domains that need complex treatment programs.

### 3.1 Study design

To collect these data, we created a private, moderated ARC hosted on Slack (Gofine and Clark, 2017). The ARC structure allowed licensed SLPs to participate at their own pace while contributing to both structured activities and open-ended discussions. The community was active for four consecutive weeks, during which participants engaged in weekly survey activities and discussion prompts. This approach balanced flexibility with continuity and encouraged both reflection and peer interaction modeled after previous ARCs (Michelson et al., 2021).

Each week was themed around an SLP practice focus, spanning from session time/frequency to facial expressiveness, and ultimately to SAR application prospects. As a primer to reflection, the participants were introduced to sample videos or articles of state-of-the-art SARs, i.e., Moxie, Misty, or other SARs used in ASD therapy research as its the most similar environment to an SLPs experiences. These are samples that were chosen to align with the topics of the week, providing participants with a foundation in the technological possibilities as well as space to consider how such systems could be integrated into their own sessions.

**FIGURE 1:**
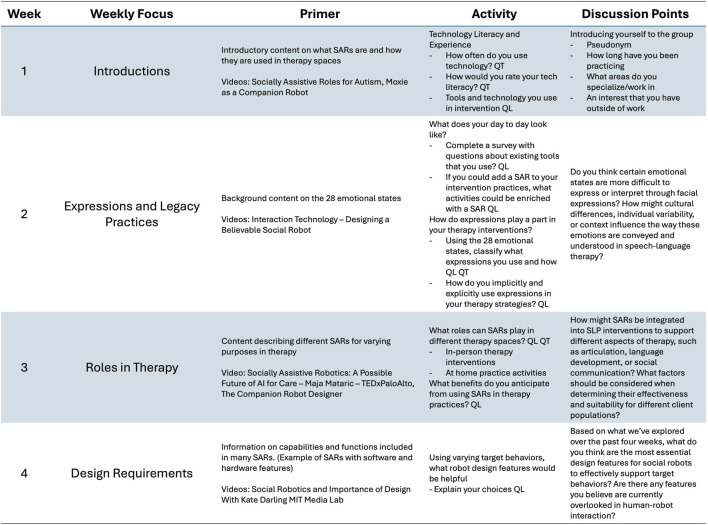
Each week participants completed a series of surveys and discussion points. This table summarizes the weekly focuses, background content, activity descriptions, and questions for discussion. QL signifies qualitative questions and QT signifies quantitative questions.

SLPs completed a mix of quantitative questionnaires and open-ended discussion questions, frequently answering in terms of their daily tasks, therapy activities, and possible use of SARs in various therapy roles. As an example, in Week 2, participants were prompted to keep a reflective note of their utilization of facial expressions and other non-verbal communication in therapy, mapped to the 28 emotional states (Cowen and Keltner, 2019). A summary of the weekly focuses and prompts can be seen in Figure 1.

Every prompt was presented with respect for the clinician’s time and preference, enabling responses on a flexible basis, with no need for synchronous presence. The ARC also placed a premium on participant comfort and ethical transparency. Weekly prompts set expectations for time commitment (usually 20–30 min per prompt), format of content (written reflection or short survey), and boundaries around sharing (no client-identifying information). Participants were invited to post publicly within the ARC platform or respond privately, fostering community and maintaining individual autonomy. This ARC study facilitated rich, grounded response that reflected both SLP practice constraints and innovative potential for integrating robotics into therapy routines. The format was especially successful at drawing out both pragmatic concerns, e.g., session timing or child engagement, as well as speculative design ideas grounded in therapeutic practice. As such, ARCs offer a replicable model for inclusive, clinician-centered co-design in emerging therapeutic technologies.

All participants were compensated $50 per completed weekly activity, for a maximum of $350. The study was reviewed and approved by the University of Nevada, Reno Institutional Review Board^3^.

#### 3.1.1 Activity prompts and discussion questions

Each week’s activities focused on a specific theme aligned with one or more research questions. Activities included a combination of quantitative survey items and qualitative prompts requiring reflective written responses. Participants were encouraged to share clinical experiences, preferences, and perspectives on how SARs might be implemented in their practice. Figure 1 summarizes the weekly focus areas, corresponding activities, and research questions addressed.

In parallel, participants engaged in open discussion threads on Slack, where the research team facilitated peer exchange, clarified prompts, and responded to participant questions. Discussion points were designed to provoke reflection, surface differing opinions, and add depth to survey responses. Figure 1 outlines the discussion topics by week.

#### 3.1.2 Thematic analysis

We analyzed weekly activity response and asynchronous discussion qualitative data using Thematic Analysis (TA) based on Braun and Clarke’s six-phase procedure (Braun and Clarke, 2006). All open-ended responses, Slack discussions, and reflection prompts were exported, anonymized, and read independently by two researchers to gain familiarity with participant language and nascent patterns. Employing an inductive strategy, semantic units of meaning were manually coded in shared spreadsheets, with multiple codes per response permitted where ideas converged. Related codes were clustered into higher-level categories via affinity diagramming (Miro), revealing emergent themes of trust in SARs, expressive fidelity, and cultural sensitivity. Themes were checked for coherence, distinctiveness, and fidelity to the raw data, then named and defined with representative quotes situating SLP practice contexts and SAR design implications. Refined themes were integrated into the Results and Discussion, supported by illustrative excerpts and, where relevant, combined with quantitative findings to present a mixed-methods view.

## 4 Results

**FIGURE 2:**
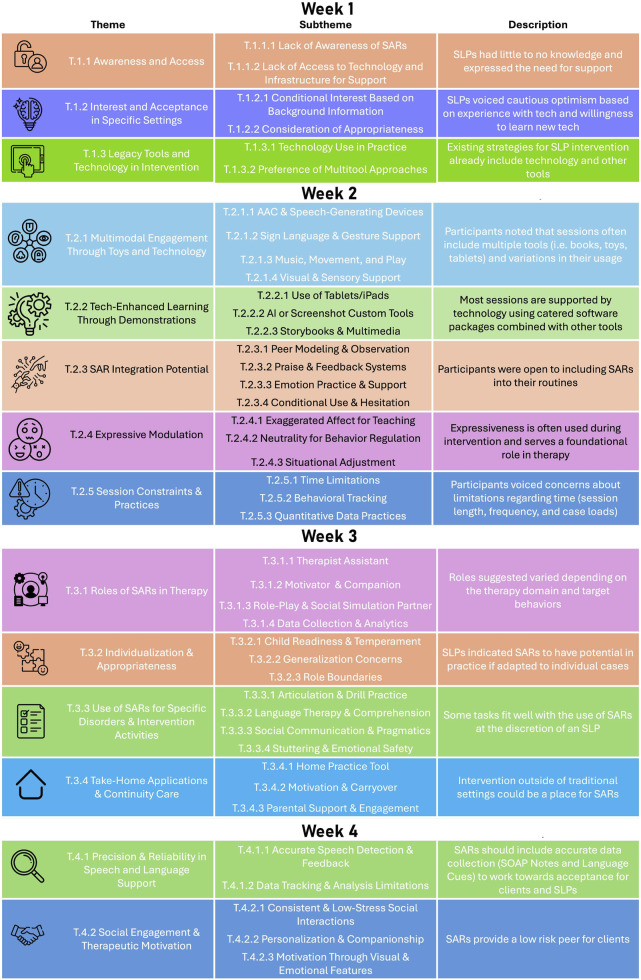
The thematic framework summarizes major themes and subthemes through out the 4-week study. This figure illustrates the layered relationship between design considerations, therapy roles, communication modalities, and system-level access.

We developed themes for each week composed of individual sub-themes. The themes are summarized in Figure 2.

### 4.1 Week 1: clinician backgrounds and insights

#### 4.1.1 Lack of awareness of SARs

A significant number of participants reported having little to no experience with SARs in any context. Participants mentioned that they had never heard of SARs before this study. One SLP shared, “I have not been exposed to socially assistive robots before today. I am certainly interested in learning more about them,” which shows a blend of curiosity and unfamiliarity. This lack of knowledge indicates that SARs are still pretty much missing from the typical speech-language therapy scene.

What’s particularly interesting is that a majority of these participants already use various technologies in their sessions. This contrast suggests that SARs haven’t quite found their place alongside tools like iPads or AAC devices. It also highlights the need for outreach, education, and demonstrations to showcase the potential of SARs as crucial first steps toward their adoption. Without some initial exposure, clinicians can’t start to assess how well SARs might fit into their therapeutic practices.

For majority, this survey or discussion was their first introduction to SARs. This really emphasizes the importance of spreading the word in ways that go beyond just academic articles, reaching out to practicing clinicians through workshops, continuing education, or hands-on demonstrations. Closing this awareness gap is key to encouraging deeper exploration or pilot programs in real-world settings.

#### 4.1.2 Lack of access and infrastructure

Even among those who showed interest, the majority of participants pointed out the systemic hurdles present in their workplaces. A number of them highlighted the absence of proper technological infrastructure as a significant obstacle. One clinician remarked, “Our department can’t even keep up with up-to-date technology,” while another noted that SARs would be appreciated “if it were handed to me, but it would be just one of many tools in my toolkit.” These insights reveal the resource limitations that are deeply embedded in institutional support and budget constraints.

The issue of access was particularly pronounced in school environments, where purchasing decisions often lie with the district and involve lengthy approval processes. Clinicians in these settings mentioned that even beneficial technologies can be underused due to issues with maintenance, training, or costs. Without dependable access to essential tools like tablets or working smart boards, the incorporation of robots is dependent on access and support from school systems or private practice.

Additionally, the feedback indicates that clinician interest isn’t the main barrier. Instead, it’s the readiness of the system and the technical support available that determine what’s feasible. Participants voiced worries not just about acquiring SARs, but also about how to maintain, update, and effectively use them. For successful integration, it’s crucial to tackle these structural challenges alongside clinician training.

#### 4.1.3 Conditional interest based on evidence

While participants weren’t quite familiar with SARs, most showed a genuine curiosity and a cautious willingness to explore their potential uses. This openness often came with a caveat: they wanted more information or proof that these tools actually work. One participant shared, “I do, however, use an iPad regularly. I do have some uncertainty but am open to learning more about them and how to utilize them.” This reflects a common theme of conditional acceptance: clinicians seem not against new tools, but they need solid reasons and real-world examples to feel confident in the integration of SARs.

Clinicians often pointed out that seeing SARs in action—whether through videos, case studies, or pilot trials—would really help persuade them. SLPs expressed expectations that hands-on demonstrations or scenarios specifically designed around speech and language goals would boost their confidence in using SARs. They stated the importance for clear links between how SARs function and the tangible outcomes for their clients.

This demand for evidence is supported by the larger framework of evidence-based practice in speech-language pathology. Clinicians are trained to assess tools based on how they impact outcomes. So, the acceptance of SARs will likely depend on clear, practice-focused research being shared. For developers and researchers, this is a call to showcase SAR capabilities in a way that aligns with the everyday demands of therapy through collaborative design.

#### 4.1.4 Consideration of appropriateness

SLPs indicated that the use of a SAR would really depend on how the SAR is situated in that treatment environment. Participants clearly articulated that they would use SARs but only if they fit the needs of their particular client and treatment goals. One participant stated, “It feels like it would be interesting to use it for helping imitate skills”, which points to the potential SARs could have for skill development. Others felt that a SAR would only be used if it aligned with the session goals of the treatment plan and the client’s readiness.

Participants expressed a strong want for customization and personal control, with the common comment being, SARs need to be customizable to the age or ability level of the client, and the behavioral profiles. They also questioned some very pertinent matters about how engaged that would need to inform their development. Instead of foregrounding technological innovation, SLPs’ clients will be, whether the skills addressed in treatment will be able to be generalized, and how to strike a balance between new and distracting. For some populations, factors such as distractibility and sensory sensitivities could cause SARs to be ineffective and present more challenges without proper adaptation.

These findings show that clinicians are taking great care when adopting technology innovations. Their decisions to adopt new tools are made with strong ethical considerations as well as considerations for efficiency. Instead of implementing the newest and trendiest tool, they are seeking to provide care that is most individualized and aligned with their approaches to interventions. Developing SARs in this space will need collaboration to learn these nuances, working to support clinicians with the development of tools that fit seamlessly into therapy with operational guarantees.

#### 4.1.5 Technology usage in practice

Even though SARs may have been a new concept for most of the participants, digital tools of one form or another were said by the majority of respondents to be used on a day-to-day basis in the sessions. These included iPads, AAC devices, educational apps, and visual supports like customized PowerPoint presentations or even YouTube videos. One clinician remarked that they had created a semi-robotic interaction system by combining the outputs of LLM with visual prompts: “I have started to build a speech robot on my own using AI tools and a screen to show prompts.” This indicates that not only does the average therapist have a comfort level with technology, but some of them have even felt the need to go one step further and innovate beyond what is currently available.

The answers clearly show the technological context into which many therapeutics are slotted. Tools are deployed to increase engagement, support multimodal learning, and ease data collection. This presents potential space for the integration of SARs, assuming that such integration does not disrupt existing workflows. A certain comfort level with screens, prompts, and programmable sequences should help place SARs within the realm of the familiar.

Additionally, clinicians noted that their use of technology was client-specific. For example, some used apps to support speech production while others relied on visual timers to support transitions or regulation. This variability reinforces the need for SARs to be customizable and modular, allowing therapists to align robot behavior with session-specific goals.

#### 4.1.6 Preference for multitool approaches

Rather than viewing SARs as a singular solution, most participants indicated that SARs would be best integrated as part of a broader therapeutic toolkit. As one clinician put it, “a robot would be one of many tools in my set.” This reflects a pragmatic approach to therapy where clinicians are used to assembling diverse strategies and tools depending on session goals.

Therapists explained that even with effective technology, flexibility is key. A tool that works well in one moment may not be ideal in the next, depending on mood, attention, or activity type. Therefore, SARs would need to adapt to this clinical ebb and flow, serving as reinforcement in some moments and stepping back in others. Participants emphasized that robots must complement—not compete with—existing methods.

Some clinicians even raised concerns that too much dependence on a single tool, especially one as visually and socially salient as a SAR, could reduce therapeutic flexibility. To be truly effective, SARs would need to integrate with existing digital ecosystems and be easily toggled, reprogrammed, or paused. The emphasis here is on coexistence, not replacement, highlighting SARs as part of an agile and therapist-controlled therapeutic environment.

### 4.2 Week 2: expressions and clinical practices

These sub-themes describe observations based on the participants’ clinical practices.

#### 4.2.1 AAC and speech-generating devices

AAC (Augmentative and Alternative Communication) systems, particularly Speech Generating Devices (SGDs), have developed to be important tools for providing support for children who have limited or emerging verbal abilities. Professionals indicated the use of both low- and high-tech AAC devices, tailored to individual requirements of each child. Numerous examples reported the use of dynamic display devices with symbol-based interfaces through which children could make requests, provide comments, or participate in turn-taking interactions. These tools not only enable expressive communication but also facilitate understanding by providing children with both visual and auditory support. One SLP reported that materials they utilized comprised “preferred activities, speech generating devices (e.g., Big Mack, iTalk2, Go Talk),” demonstrating the variety of methods of AAC implemented in practice.

Ordered routines also included SGD use, in the form of toy-based play, with prompts as appropriate to the child’s level of independence. Most SLPs indicated a focus on instructing core vocabulary (e.g., “go,” “more,” “want”) to enhance functional communication skills. One SLP elaborated that “during the session, the [client] will request “more” (e.g., of favorite song or toy) using AAC,” showing the explicit use of communication objectives with AAC devices.

Despite their benefits, SGDs also presented challenges. Some SLPs noted that device calibration or malfunctions could interrupt the flow of therapy. Others raised concerns about over-reliance on technology when symbolic understanding or motor planning was limited. Still, clinicians saw SGDs as empowering tools that could give voice to children who would otherwise struggle to express themselves. As one SLP explained, modeling stop via AAC. The student will then request again if necessary,” illustrating how AAC can support both regulation and agency.

SGDs were often incorporated into multi-sensory learning approaches. The practitioners stressed the importance of combining auditory and tactile input through the use of music, visual, and body contact to captivate the learner. One practitioner commented as follows: “the target behavior is to use multimodal communication (e.g., AAC, vocalizations, gestures) to request songs/activities.” In addition, tools like iPads and tablets were referred to as important AAC channels within play-based or sensory-integrated intervention. For example, a participant quoted how the “iPad … music can be a basic cause and effect activity to engage,” showing the role of digital AAC devices in maintaining attention and provoking communication.

#### 4.2.2 Sign language and gesture support

SLPs frequently described the use of gesture and gestures as adjuncts to verbal and AAC communication. The modalities were typically presented as precursors to more expressive powerful devices, especially for beginning communicators. Gestures provided a visual, kinesthetic means of communicating basic needs such as requests, refusals, or naming actions. Because one participant depicted nonverbal communication as “facial expressions, gestures (pointing), expressive motions e.g., (“more”, “help”).”

A number of therapists described that gesture use extended beyond the formal sign systems like ASL. Pointing, waving, or using body movements were all interpreted as purposive tools in therapy. The gestures helped children achieve shared attention or task progression. One SLP explained, for example, “For mainly articulation, some hand gestures can help with sounds and they can often associate a hand gesture with a sound.”

Therapists recognized that the success of this approach was contingent upon motor skills and imitation skills. For kids with severe physical disabilities, sign approximations or more simplified gestures were encouraged. As stated by an SLP “Some kids can’t make the full sign for “help”, but their approximations still get the message across and are celebrated.”

#### 4.2.3 Music, movement, and play

Music, movement, and play were highlighted as a means to enhance engagement and facilitate regulation. SLPs described using songs, rhythmic activities, and gross motor play as means to facilitate communication goals. Ball poppers, stuffed animals, or books were utilized not just as sensory input devices but as a means to elicit requests, joint attention, and vocabulary. These activities were also structured with clearly defined routines that provided predictability. One clinician shared, “To engage the student and get the desired behavior I use toys such as a ball popper, bubbles, and critter clinic. All of the toys have cause and effect, so students are kept engaged and will request.”

Music specifically was prized for its universal interest and predictability of rhythm. Therapists employed known melodies to facilitate transitions, build up desired sounds or words, or prompt motor imitation. For children with minimal verbal communication, clinicians illustrated how rhythmic clapping or dancing could serve as a means of expression or as a turn-taking cue. As one participant noted, “tools used are AAC device and music player (iPhone or iPad). The music can be a simple cause and effect toy (music on, music off).” Music was also used to regulate states of arousal, specifically in dysregulation-prone clients.

Play was placed as both a process and end of engagement. With fantasy play, such as pretend cooking or animal play, the SLPs modeled sentence structure, verb use, and social interactions. With younger clients, play enabled shared attention and symbolic representation. Older children utilized structured games to address turn-taking or conversational reciprocity. Play was also individualized: what was stimulating to one child (e.g., kinetic sand) might be overstimulating to another. One SLP stressed, When playing, I withhold the barns so that the child may ask for them. The child communicates with me by requesting.

During these interventions, therapists emphasized the importance of reading the child’s affect and rhythm. When a child became disengaged or overstimulated, clinicians changed the rhythm or modality. This flexibility allowed therapists to balance excitement with calm and to offer intervention objectives in developmentally appropriate, client-centered fashion. As one counselor explained, “They either use functional or make-believe play or they play with the items. Depending on the child’s attention, they usually interact with me when they need to ask for something or need assistance.”

#### 4.2.4 Visual and sensory support

Therapists consistently referenced the use of visual and sensory tools to support communication, regulation, and engagement. Visual supports included picture schedules, token boards, and emotion cards, which were particularly effective for children with autism or processing challenges. These tools helped clarify routines and expectations, reducing anxiety and promoting independence. As one therapist described, reviewing visuals was a foundational step: “The first thing I do in every session is review the visual schedule—it sets the tone.”

Visual representations of emotions were utilized to identify and communicate emotions, sometimes embedded within social-emotional learning curricula. Practitioners reported activities in which children chose images corresponding to their emotions or identified how a character might be feeling in a story. These materials facilitated word building and the construction of perspective-taking abilities. As one practitioner clarified, “I use a lot of pointing. Sometimes it is helpful to provide a visual cue when there isn’t a verbal cue.”

In terms of sensory interventions, a variety of weighted blankets, fidget tools, textured mats, and swings were used both proactively and reactively. When a student became dysregulated, these tools were implemented to assist in regaining attention and emotional state. For instance, as an example, one of the therapists explained that “tools used are toys that are engaging and themed based, such as barn with animals, animal puzzle, animal flashcards.”, illustrating the notion that sensory support can provide an environment that is welcoming to connection and communication.

Specifically, the therapists indicated the requirement for individualizing visual and sensory supports. While one child would find certain stimuli calming, another would be overstimulated; thus, visuals must be developmental stage-specific and applied consistently. By observing carefully and modifying these tools, clinicians rendered access and participation very responsive.

#### 4.2.5 Use of tablets/iPads

The most frequent report by SLPs was the use of tablets and iPads as invaluable tools in supporting both communication and participation in therapy. These devices were particularly useful for incorporating interactive apps such as articulation games, sentence-building tools, or video modeling sites. Therapists indicated that the touchscreen interface and animated graphics had a tendency to increase attention, especially in children who would otherwise be distracted or disengaged.

Apps were chosen with care to support therapeutic objectives. Ultimate SLP^4^, for example, was utilized to allow practice of targeted sounds or themes, while some were chosen due to their flexible communication boards that could be utilized to represent a child’s daily schedule. Several therapists explained how digital tools permitted more flexibility and personalization, tailoring the content to the client’s language capacity, interests, and sensory profile.

Beyond their use for structured activities, tablets were used as vehicles for narrative development, task sequencing, and monitoring of behavior. For some clients, the devices were utilized as rewards or incentives, while for others, they represented a favored method of self-expression. Clinicians were sensitive to screen time and frequently combined tablet use with offline activities to encourage learning in a multimodal fashion.

Some therapists expressed concerns about excessive reliance on technology or difficulties in managing expectations if a session did not include iPad use. Others also underscored the need for proper training and problem-solving, especially when technical issues halted the course of the session. However, when used mindfully, tablets were seen as powerful tools that combined therapy practices with play interventions.

#### 4.2.6 AI or screenshot custom tools

The utilization of artificial intelligence-driven websites and personalized screenshot software to develop tailored therapeutic materials emerged as a sub-theme. A few practitioners reported utilizing image capture programs or generative text algorithms to produce social stories, behavioral images, or prompt-response cues. The digital programs enabled the development of very personalized materials that corresponded to the child’s specific settings, routines, or emotional requirements.

Screenshot software was frequently utilized to capture the daily activities, settings, or objects that the child was exposed to. For instance, a therapist may photograph a school hallway to facilitate transitions or create a visual script with real photos from the student’s daily life during the course of the day. This type of visual identification served to alleviate anxiety and enhance contextual salience.

Artificial intelligence was also used to generate examples or ideas for lesson planning. Some therapists explored using chatbots or text generation programs to generate question banks, complete emotional check-ins, or develop articulation sentences. While this use was still in an experimental phase, it represented a growing interest in using technology to reduce preparation time and improve creativity.

Despite the promise, clinicians also noted constraints such as privacy concerns, device availability, and having to learn new technologies. Additionally, not all clinicians were at the same level of expertise or training in using these tools. But early adopters noted a great deal of potential to create tailored, accessible content that could improve therapy outcomes.

#### 4.2.7 Storybooks and multimedia

Storybooks and multimedia resources remained a part of therapies. Digital storybooks or YouTube videos were used by the participants to present topics, demonstrate how to build sentences, or facilitate discussion. These media offered a structured and interesting format that fostered vocabulary and story-telling abilities.

Digital storybooks were particularly useful for kids with limited attention spans. By including auditory effects, animated sequences, and visual prompts, these tools made storytelling more interactive. Practitioners often paused the narrative to ask questions, solicit predictions, or model vocabulary for emotions. In some cases, multimedia books were paired with manipulative story aids or sequencing cards to support comprehension.

Multimedia materials were also used to teach social-emotional skills. Clips of children resolving arguments on the playground or becoming frustrated were used as discussion starters. Therapists reported using the technique of stopping videos to discuss facial expressions, tone of voice, and problem-solving strategies. This audiovisual approach allowed children to connect words with what happens in their lives.

However, clinicians were careful in selecting media and screen time. They preferred short, targeted videos or interactive e-books over passive viewing. They also incorporated follow-up activities such as drawing, retelling, or acting out portions. Multimedia in general was valued for its ability to engage, inform, and support richer learning.

#### 4.2.8 Peer modeling and observation

Therapists consistently emphasized the value of modeling as a vehicle for communication development—whether through adult demonstrations, other children, or pictures. SARs are particularly suited to act as consistent, available peer models in these environments. Because human peers are less easily in a position to model target behavior with impeccable consistency, robots can be programmed to offer children predictable models of core vocabulary, AAC, or turn-taking routines.

Practically, this might mean having a SAR model press a communication button to say “go” and then wait for the child to imitate. For group therapy, SARs can be a peer who always shares, waits his turn, and greets with enthusiasm—behaviors that therapists often try to scaffold children into doing through modeling and reinforcement. Their ability to repeat these behaviors again and again makes them a good fit for observation-based learning, especially when human peer models are inconsistent or absent.

Furthermore, therapists noted that some children showed greater interest when a child modeled a skill. SARs can simulate this social learning dynamic under less pressure, offering children a “social partner” that is engaged but not harried, spanning observational learning and interactive play.

#### 4.2.9 Praise and feedback systems

Praise and feedback were consistently mentioned as methods to reinforce communication attempts, persistence, and engagement. SARs can be programmed to deliver immediate, affectively rich feedback using visual gestures, lights, facial expressions, and speech. This multimodal response system reflects therapist techniques but can sustain a degree of consistency and novelty hard for humans to match session after session.

For instance, a robot may react to a successfully fulfilled request by expressing a jubilant “Nice asking!” while simultaneously exhibiting a smile and clapping gesture. Gradually, this form of positive reinforcement can enhance the child’s self-assurance and drive. Numerous therapists have observed that the enthusiasm and emotional energy displayed by adults play a crucial role in maintaining engagement during sessions—SAR with lively responses can effectively imitate this emotional responsiveness in a sustainable and non-exhaustive manner.

SARs also possess the ability to deliver structured corrective feedback in an affectively neutral manner. When the child selects an erroneous symbol, the robot can politely guide them to try again or model the right one. Because the feedback is not presented through human judgment, it may possibly be less stressful or punitive to the child, especially anxious or sensitive children. With this combination of praise and redirection, SARs are promising feedback agents for varied therapeutic tasks.

#### 4.2.10 Emotion practice and support

Therapists often included emotion recognition and regulation in treatment. SARs can assist these objectives by modeling emotional expressions, identifying affective states, and leading children through co-regulation activities. Their programmable expressions—on-screen faces, gestures, or audio tones—can help make abstract feelings more tangible and practicable for children with difficulties in emotional literacy.

For example, a SAR may say, “I am sad when my block tower falls,” with a frowning face and slouching posture. This kind of modeling facilitates emotion vocabulary acquisition and language linking to body sensations in children. Robots can also help children select their own emotion on a chart or act appropriately to social stories—offering the opportunity for identification along with empathy development.

Under conditions of dysregulation, SARs can serve as co-regulators, initiating calming routines like slow breathing or listening to calming music. A few therapists mentioned the importance of pacing, arousal management, and affect-responding adaptivity; SARs can enable such adaptivity by virtue of simple emotion-based routines. While they do not replace human emotional presence, the ability to offer stable, non-reactive support can enable the creation of a calming and responsive space.

#### 4.2.11 Conditional use and hesitation

While the majority of therapists were enthusiastic about technology-enhanced methods, some also reported reluctance towards overuse or misplaced matching with client needs. SARs must be used judiciously, with consideration for when and how they add to—instead of subtract from—the therapeutic process. That is, identify which children are helped by SARs and which will find them distracting, confusing, or over-stimulating.

Some participants noted that children with high sensory sensitivity or rigid preferences may reject the robot or become overly fixated on the robot in ways that detract from session goals. SARs thus must be implemented conditionally, tailored to accommodate individual response, and augmented with familiar tools and routines. For instance, a child may perform optimally with SAR implementation only during transition, whereas another performs optimally with robot-mediated AAC modeling throughout.

In addition, there were concerns from some therapists that the novelty of SARs would be lost or create dependencies. To prevent this, robots must be viewed not as substitutes for human connection but as strategic augmentation—tools to offload mundane modeling, underscore consistent language, or aid in co-regulation. Conditional use guarantees SARs are implemented ethically, responsively, and in service to the child’s needs.

#### 4.2.12 Exaggerated affect for teaching

Therapists frequently use exaggerated facial expressions, vocal inflection, and gestures to emphasize key ideas and draw children’s attention to important social or linguistic cues. This “performative” affect helps clarify emotion vocabulary, increase engagement, and reinforce prosodic features in speech. Participants shared that they rely on animated facial expressions—big smiles, gasps, or frowns—to cue reactions, build excitement, or scaffold comprehension during stories, routines, or games.

SARs can simulate exaggerated affect with expressive facial displays or entrained movement. For instance, a robot can raise its eyebrow and widen its eyes and say, “Oh no!” when confronted with a virtual spill. Such repeatable, high-contrast expressions allow young children—especially children with developmental delays—to link emotional meaning to visual and auditory input. Exaggerated affect in therapy is not just performative; it is pedagogical.

When carefully programmed, SARs can take this strategy one step further by providing repetitive, expressive demonstrations that never tire or lose sight of the desired model.

Hyperaffect can also serve as an anticipatory cue. A gasp of surprise or a wide-eyed pause might be used to signal that something is going to take place, with the child reacting or listening. SARs that can elicit these affective reactions can thus facilitate pacing, attention, and comprehension in the course of early intervention sessions.

#### 4.2.13 Neutrality for behavior regulation

While affect is often exaggerated to teach or engage, clinicians also described the importance of remaining neutral in moments of dysregulation or challenging behavior. A calm demeanor, steady voice, and limited facial expression can help de-escalate children who are overwhelmed, frustrated, or overstimulated. This “affective neutrality” is especially critical in maintaining safety, redirecting behavior, or offering consistent boundaries without escalating tension.

SARs are particularly well-suited for delivering this kind of calm, neutral support. Unlike humans—whose tone or expression may subtly change due to fatigue, frustration, or stress—robots can maintain a steady affect across situations. For example, if a child throws an object or begins to cry, the SAR might respond in a measured tone: “Let’s take a breath,” accompanied by a neutral expression and slow gesture. This lack of reactivity can reduce emotional contagion and support co-regulation.

Therapists also reported that children sometimes escalate when they sense emotional tension in adults. In contrast, SARs offer predictability and affective containment that may feel safer or more manageable to children. This neutrality doesn’t imply coldness—it means emotional consistency that supports regulation without overstimulation.

#### 4.2.14 Situational adjustment

SLPs noted that they constantly modulate their emotional tone in accordance with the child’s affective state, activity context, and therapeutic goal. They can intensify energy to match a child’s excitement or lower their tone to help calm a distraught or dysregulated child. This fluid management of emotional expression relies on sensitivity, timing, and relationship awareness—all of which are the building blocks of therapeutic attunement.

In order to successfully employ SARs in therapy, they must replicate this flexibility. Since most robots operate on pre-scripted affective behavior, advanced SARs may alter tone, volume, gesture speed, or facial expression based on contextual cues—manually prompted by the therapist or, eventually, gleaned from sensors (e.g., motion, gaze, or vocal data). A robot, for example, may transition from a high-energy music routine to a softer-voiced bedtime routine when a child is upset.

Therapists also expressed the use of affect to signal activity transitions: cheering when a task is finished, stopping with a solemn tone prior to delivering a rule, or assuming a nurturing tone when presenting something new. SARs that are able to include this kind of contextual affect switching will be more engaged in the relational rhythm of therapy. While true emotional attunement is a unique human ability, SARs that are able to modulate situational expressiveness can still provide more developmentally congruent support and smoother transitions.

#### 4.2.15 Time limitations

Time limits were cited universally by clinicians as a key factor in planning and implementing sessions. With sessions being typically only 30 or 60 min long, therapists reported having to make hard choices about which goals could be addressed first. Participants mentioned that routines, AAC system setup, and transitioning on a regular basis took up a lot of available time with less space for dense language modeling or child-led activity.

It was mentioned by some respondents that certain activities were occasionally abbreviated due to late arrival, emotional dysregulation, or consultation with caregivers. Such time limitations typically led to a more prescriptive or structured methodology, with no room for unstructured play or emotional exploration. One respondent discussed the need to “front-load high-priority targets” at the start of the session, with an awareness of not being able to cover all intended targets. Some respondents described using pre-established routines in an effort to boost efficiency and reduce transition times.

In spite of these time pressures, healthcare professionals stressed the need to retain moments for interaction and bonding, despite the time constraint. Several solutions were proposed through strategies such as reducing the content to be covered, making transitions smoother, or integrating several objectives into a single activity (e.g., motor play that also fosters vocabulary development or self-regulation). These solutions mirror the creative balancing act that therapists undertake to optimize limited time while continuing to address the child’s needs.

#### 4.2.16 Behavioral tracking

Both observation and data collection were cited as crucial, albeit challenging, aspects of session activities. Practitioners indicated they employed various methods, including tracking forms, tallies, or applications, to record behaviors such as request frequency, successes with specific targets, or dysregulation instances. Practitioners did, however, recognize the issues inherent in gathering data at the same time as conducting interactive, relationship-based interventions. For some, having to “capture everything” hindered the spontaneity of their interactions.

Several therapists commented that some behaviors—particularly those concerned with emotional regulation or social reciprocity—were hard to measure in the moment. Some practitioners also mentioned dependence on retrospective recording, usually undertaken at the close of the session or end of the day. Delays of this nature can result in possible inaccuracies or lack of picked-up subtleties in behavior trends. Notwithstanding these difficulties, clinicians appreciated behavior monitoring as a means of evaluating change, spotting patterns, and guiding future planning.

Therapists also described collaborative monitoring with families, more so in home or telepractice settings. Sharing charts or asking caregivers to mark behaviors, some clinicians extended observation beyond the session. However, participants acknowledged the limitation of this practice, especially when caregivers were stressed or new to data recording protocols. These remarks reflect the tension between the ideal of constant monitoring and the pragmatics of a relational therapy setting.

#### 4.2.17 Quantitative data practices

Quantitative data recording practices like percent accuracy, frequency, and trial-based data were frequently mentioned as a requirement of accountability, especially in school and insurance-based settings. Several clinicians reported accounting for discrete skills such as requesting, labeling, or matching on structured tasks, with some indicating the use of pre-printed data forms or rubrics. These kinds of data were utilized to demonstrate progress, justify ongoing services, and guide goal revisions. This subtheme also supports themes found in later weeks such as Section 4.3.4 and Section 4.4.2.

Simultaneously, practitioners also expressed concern over the simplification of rich behaviors into numbers. Some worried that strict data collection procedures might obscure significant, yet less quantifiable, progress—such as more eye contact, spontaneous initiation, or emotional display. Some were also concerned that ongoing quantification might get in the way of rapport establishment or responsiveness, particularly in play-based or emergent communication interactions.

A few participants discussed altering quantitative methods to be a better fit for their clients. This was sampling rather than continuous recording, rating scales rather than binary scoring, or functional outcomes rather than mastery of discrete trials. SLPs mentioned that formal data needed to be counterbalanced with narrative notes and clinical judgment, especially for children whose progress was neither linear nor readily quantifiable.

### 4.3 Week 3: roles in therapy

#### 4.3.1 Therapist assistant

Clinicians commonly envisioned SARs as tools that would be capable of supporting session flow and reducing clinician burden. Participants described situations in which a robot could deliver consistent prompts, model desired behaviors, or remind children about activities while the therapist worked on other demands. These assistant roles included managing transitions, leading structured routines (e.g., warm-ups, games), and prompting children to respond or take turns. Some therapists imagined SARs initiating a song or showing a visual schedule at the start of a session, offering a predictable structure for clients.

Therapists made it clear that although SARs might serve as valuable assistants, their presence ought to be optional. For instance, one clinician wrote that “a robot could help with prompts, cues, visuals, and scaffolds (possibly at the verbal request of the clinician). Once the clinician makes the judgment call it is quite reasonable for the robot to carry out the execution at command.” This is a shared-control model in which clinicians are still in charge but can delegate routine actions to the robot. SARs were also reported to be handy in managing group routines or activities—particularly where therapists had to monitor or adjust strategies in real-time. One respondent noted, “a robot could help with role-playing social situations with myself or the students,” thus highlighting the flexible and supportive extent of the robot’s use.

#### 4.3.2 Motivator and companion

Another common theme among responses was that SARs could enhance motivation and interest, particularly for individuals who fatigue easily or object to repetition. One clinician commented that “robots would be amazingly fun for the kids and could be used to facilitate participation,” in that SARs could potentially make otherwise monotonous activities more game-like and fun. Others stressed that a robot could work as a “buddy” or “companion” to establish a social presence that interacts over the long term.

The fact that the children liked new or interesting technology was considered an advantage, with one other therapist commenting that “a robot could potentially teach a child. Giving them cues as to how to correctly produce speech sounds,” particularly if paired with a reward or incentive system. Some even saw SARs delivering music or a dance reward upon completion. The robot’s presumed happiness and neutrality were seen as ways of giving children the external motivation they needed to push through boring or hard tasks.

#### 4.3.3 Role-play and social simulation partner

SARs were widely perceived by clinicians as ideal partners for role-playing social interactions in low-stress, predictable ways. Clinicians saw potential in SARs participating in greetings, turn-taking exercises, or fantasy play, with children being given a non-critiquing and predictable model of interaction. A clinician explained that “robots would be great at role-playing because it would alleviate the stress of having to communicate with actual people, but still gain practice,” suggesting that SARs could be leveraged to help calm children who are overwhelmed by interacting with humans.

One therapist noted that SARs “can provide a consistent and non-judgmental presence,” which is especially helpful for clients who struggle with peer rejection or fear of being wrong. This capacity to simulate interactions in a consistent manner was seen as one of the strengths of SARs in building social confidence and scaffolding to more unpredictable, human communication partners.

#### 4.3.4 Data collection and analytics

SLPs also expressed a keen interest in utilizing SARs as data collection and documentation tools, especially in environments where monitoring and participation are challenging concurrently. One therapist explained how, “if the device itself recorded data. That would free up the clinician to be more involved in the activities and not tied to taking quantitative measurements,” expressing a wish to reduce the administrative load of tracking progress in the moment.

Others suggested SARs could record behavioral measures like response time, success of turn-taking, or task completion, either alone or in concert with therapist input. Participants cautioned, however, that “data collection may be hard for things like articulation because at times it takes an ear of keenness to detect distortions,” and SAR-collected data should therefore be secondary and interpreted contextually. Others commented on the value of longitudinal data gathered by SARs for identifying patterns across sessions.

#### 4.3.5 Child readiness and temperament

Clinicians stressed the importance of assessing readiness on a case-by-case basis before introducing SARs. While one SLP noted that “some would be keen on the robot and want to do it,” others warned that “some will be resistant or even scared.” One therapist said, “it may be difficult to get some students to focus on the robot. Others may do it too much,” leaving open the possibility of both avoidance and over-fixation.

Therapists emphasized matching SAR characteristics—like voice, animation, or movement style—to children’s sensory profiles and preferences. These responses placed SARs in the realm of potentially useful, yet only if applied intelligently and diversified for each child’s unique behavioral profile. As one therapist clarified, “I have some kids who would respond to the predictability and structure, and others who would shut down completely if something unknown walked into the room.”

#### 4.3.6 Generalization concerns

Several SLPs were worried about whether or not skills practiced with SARs would generalize to human interaction. A therapist asked, “The child might do great with the robot, but will it transfer to another child in the playground?” Another commented that “it might be easier to talk to a robot than to another human being. But it might not transfer to real-life communication,” especially for individuals with autism or social anxiety.

Participants recommended beginning with SARs as introductory scaffolding, in conjunction with human practice and fading techniques to maximize real-life carryover. Some clinicians noted that generalization would entail specific planning, such as progressing from SAR-mediated role-plays to interactions with friends, or incorporating the robot into human-mediated therapy routines.

#### 4.3.7 Role boundaries

A theme across responses was that SARs should never replace therapists. Clinicians concurred that robots could help, direct, or cue, but the therapist ought to retain power and relational depth. As one member clarified, “The robot is a tool. I am still the clinician.” Another echoed, “I would not want it to replace me or the relationships we build in therapy,” voicing a vision of SARs as addition, not replacement.

Therapists desired control over task pacing, affect responsiveness, and clinical decision-making. Some also mentioned the need for real-time override or flexibility: “If the student is upset, I need to be able to stop the robot right away.” These comments suggest that SAR integration must be therapist-centered, customizable, and optional within the therapy process.

#### 4.3.8 Articulation and drill practice

Some SLPs saw SARs as valuable resources for articulation therapy, especially for drill-oriented activities that necessitated repetition and consistency. The robot’s capacity to provide cues in a non-variable and non-fatiguing way was perceived as helpful in keeping the child attentive and in expanding practice opportunities. Participants explained how SARs might cue children to repeat words or phrases, reinforcing sound production with positive feedback.

However, there were also reservations. Some therapists questioned the utility of SARs for articulation without visible articulators to act as models for tongue or lip position. This limitation was particularly relevant for those sounds which rely on precise motor placement, where visual modeling by the clinician is of value.

One participant commented, “The use of robotic support may make it easier to practice activities like repetition and drill tasks for speech sound disorders, but one has to make sure that the robot doesn’t act as a distraction or limit opportunities for natural speech production.” Another participant said, “Without a visual model of the articulators, it can be challenging to teach the correct placement for certain sounds.”

#### 4.3.9 Language therapy and comprehension

Language development tasks such as answering WH-questions, sequencing, or retelling were typical examples provided as activities that SARs could support. Several therapists also mentioned that SARs could provide a lead for a story, ask basic questions, or provide visual supports while the child responded. These functions were thought to provide additional support to comprehension and expressive language practice.

Therapists appreciated the idea that SARs could deliver brief, immediate feedback and repeat prompts consistently. One participant commented, “It could be used to help facilitate story retell activities or question a story. Especially if it had a fun voice or animation.” Another recommended, “It could help by having questions and prompts presented in a consistent manner that doesn’t change or get tired.”

These considerations made SARs suitable for formal, guided language activities while maintaining the need for therapist control in more complex linguistic exchanges.

#### 4.3.10 Social communication and pragmatics

Clinicians noted that SARs would be able to assist in social goals, most prominently in initiating, maintaining, and ending interactions. With repeated, scripted interaction, SARs could help children practice turn-taking, eye contact, and greeting rituals.

One of the participants noted, “Some of my students who are learning social skills would be helped by a robotic entity that could model a social routine or offer them a stable interaction partner.” Another participant noted, “I believe they could be helpful for students who have shyness or fear. A robot could help build their comfort in practicing greetings or engaging in small talk.”

Therapists stressed that although SARs can augment practice, those aspects of generalization and spontaneity continue to need human interaction and adaptive support.

#### 4.3.11 Stuttering and emotional safety

Several participants discussed how SARs might benefit children who stutter, notably in the ability to offer a judgment-free zone for practice. One clinician commented, “It could be helpful for students who are nervous speaking in front of people—having a robot present might reduce the pressure.”

SARs were not considered explicit therapy agents for fluency shaping but rather supportive companions to practice strategies and build confidence. Another participant stated, “They would not be able to give feedback on secondary characteristics or emotional responses, but for low-pressure practice, it could help.”

#### 4.3.12 Home practice tool

Some SLPs expressed that they would be interested in having SARs as home practice partners. SARs could guide parents and children through scripted activities, go over the goals, and encourage repetition daily. This was seen as a way to extend therapy beyond clinic walls.

One participant commented, “A robot could lead the child through home practice, especially if it can be programmed by the therapist or linked with session goals.” Another participant stated, “If parents are struggling to remember what to do, it could give them cues and models without needing another session.”

The idea of an SAR-based home routine was found especially valuable for families with little time or therapeutic expertise.

#### 4.3.13 Motivation and carryover

SARs were seen to have the potential to be strong encouragements toward ongoing use of skills outside the treatment setting. As one therapist put forward, “If the robot is enjoyable and interactive, the child might request to do their homework with it—which is beneficial for all.” Another commented, “Children will more willingly work with something they perceive as a toy or friend.”

These responses highlight the perceived ability of SARs to generate carryover through novelty and fun, particularly with children who are resistant to conventional homework formats.

#### 4.3.14 Parental and engagement

Some SLPs described how SARs could act as bridges from home to clinic. In addition to facilitating child practice, SARs may show techniques or recap sessions. “Sometimes parents don’t sit in sessions and don’t know what to do—if the robot could repeat the target or show the strategy, that would help,” one observed.

One noted, “It could send a message home or display a brief clip to remind the parent what to practice.” Therapists saw this as a way of engaging caregivers without imposing, especially in under-equipped or busy households.

### 4.4 Week 4: design requirements

#### 4.4.1 Accurate speech detection and feedback

SLPs throughout emphasized that for SARs to be effective in phonological and articulation treatment, they must be able to detect subtle differences in speech. Participants spoke of the necessity for SARs to possess the capacity to judge children’s speech quality accurately. As one clinician explained, SARs would need to “properly analyze a child’s speech, identify any phonological processes or articulatory distortions/errors, and then provide suitable cues.” This kind of feedback normally relies on subtle auditory cues that clinicians learn to pick up on.

While there was excitement, most clinicians were apprehensive about the viability of such capability. Some reflected on the challenge of SARs picking up on subtle speech errors in children, such as phoneme distortions or substitutions. The concern was that if the robot couldn’t match the perceptual ability of a trained clinician, then it might be reinforcing incorrect productions or missing emerging gains. The sub-theme thus delineates both the desire for precision and the skepticism that current speech technology is ready to meet clinical levels.

#### 4.4.2 Data tracking and analysis limitations

Progress monitoring and data collection were seen as rich but complex potentialities for SARs. Participants liked the idea of a SAR that could “monitor data” such as correct or incorrect productions from trial to trial. Participants, however, expressed concerns about reliability and consistency of such monitoring, especially given the variability inherent in client speech and behavior. A clinician commented, “Numerous variables are shared across sessions,” indicating that SARs might not control for contextual factors or intention in performance ratings.

A few therapists envisioned SARs being used to collect baseline or current-level performance data, particularly if the SAR could “detect a correct vs. incorrect” and find trends over time. This would be especially useful for documentation or assessment updates. But clinicians pointed out that inconsistent or over-simplistic feedback from the SAR would interfere with the therapy process or be confusing. Generally, the introduction of data features was well received, provided that it was carried out under close supervision by a human clinician.

#### 4.4.3 Consistent and low-stress interactions

A significant benefit cited by clinicians was the potential of SARs as low-pressure, predictable social partners. Participants suggested that SARs could help in “practice building a connection with less at stake,” particularly for students who struggle with human-to-human interaction. This was most attractive to clinicians working with autistic or socially anxious clients, for whom uncertainty in social response may increase stress.

Because SARs can be programmed to respond in a predictable way, they can help clients rehearse greetings, turn-taking, or pragmatic routines in a structured manner. One participant pointed out that SARs “build confidence” through repeated, nonjudgmental correction. This makes them well-suited to early stages of social communication training, where comfort and fluency are more important than spontaneity or improvisation. While generalization to human partners is the long-term goal, SARs were seen as helpful stepping stones.

#### 4.4.4 Personalization and companionship

Therapists saw potential in SARs as personalized companions that could enhance therapy objectives along with emotionally engaging with clients. The concept of SARs being customized in appearance and function came up repeatedly, with one therapist proposing that they should permit “changing the color or light scheme of the device,” which could be used to help kids create positive associations or even mimic dress-up or customization play.

Other participants described SARs as “peers” or “friends,” especially for populations such as stroke patients or individuals with cognitive disabilities. These populations benefit from structured companionship and frequent contact. In this role, SARs are more than an instrument—they are half-social agents that maintain therapeutic rapport, routine, and regulation.

#### 4.4.5 Motivation through visual and emotional features

Clinicians stressed that SARs must not only be functional but also visually and emotionally appealing. SARs must “show facial expressions,” one therapist indicated, if they are to be of help in the development of pragmatic language. This is consistent with a widespread view that emotional responsiveness, not merely verbal response, is central to engaging interaction.

They also discussed how SARs could be used to facilitate task completion. Animated imagery, motion, and vocal inflection were listed as the critical variables in ensuring child participation. Stationary or blank-faced robots were deemed second-best. One therapist explained that SARs need to offer “consistent reinforcement, with a clear target,” i.e., cheering or flashing at a child’s achievement. These multimodal feedback techniques were thought to increase participation and aid in learning behavior, particularly with children or attention-variable patients.

## 5 Discussion

The thematic analysis provided a nuanced picture of the usage of SARs in clinical spaces by SLPs. Our findings demonstrate a cautious desire for SARs that are expressive but adaptable, customizable but standardized, and engaging but therapeutically sound. Participants emphasized, specifically, adaptive expressive capabilities, compatibility with multimodal communication, and integration with existing therapy materials and workflow. These were not regarded as add-ons, but as essential to adoption and sustained use. The findings also depict conflicts between the potential of SARs as therapeutic tools and the pragmatics of implementing them in resource-constrained, highly individualized therapy environments.

This research informs continued human-robot interaction and assistive technology development by highlighting field-based design expectations from practicing SLPs. This aligns this work with previous research in SAR design that highlights the value of emotional expressiveness and embodied interactions for facilitating engagement and social learning, especially in pediatric applications (Breazeal, 2003; Belpaeme et al., 2018). Our results extend, but complicate these findings by demonstrating that SLPs desire not just high expressivity but dynamic control over affective presentations to suit a variety of client sensitivities. This is consistent with work emphasizing the value of personalization in SAR behavior (Leite et al., 2014) and with work showing the importance of adapting robot interaction methods to individual neurodevelopmental profiles (Scassellati et al., 2012).

Similarly, the call for reconfigurable behavior modules is in line with participatory and co-design work in rehabilitation robotics that champions the need to involve practitioners in order to ensure robotic capabilities are aligned with therapeutic agendas (Šabanović et al., 2014). SLPs in our study echoed this in their demands for routines and content that could be tailored, with the concomitant call for open authoring tools or clinician-facing programming interfaces. Additionally, earlier SAR deployments in clinical and educational settings have shown that even technically feasible systems can fail due to lack of consideration for infrastructure constraints, data privacy, and professional training (Matarić et al., 2007). Our participants concurred with this assessment, citing institutional bans (e.g., camera bans, device approvals) and emphasizing the need for robots to be readily accommodated within existing therapeutic settings.

### 5.1 RQ1: What experiences from an SLPs typical routine can inform the design of a SAR?

Throughout the 4 weeks of clinician input, a number of themes arose that pertain to our main research questions: (1) What are the attitudes and expectation of SLPs regarding the use of socially assistive robots (SARs) in therapy? (2) In what ways do SLPs foresee using SARs in their clinical work? (3) What are the design and contextual prerequisites for effective SAR adoption?

SLPs’ assumptions regarding SARs were typified by general unfamiliarity, as observed in Week 1 answers. The majority had never heard of or employed SARs, but they were eager to learn more—particularly when shown evidence of impact. Numerous respondents did not perceive SARs as distractions but rather as potential therapy enhancers when used appropriately. With the progression of weekly discussions, this openness developed into more informed and better-educated expectations.

By Week 2 and thereafter, clinicians were expressing specific expectations: SARs should be expressive, affectively engaging, and able to perform standard therapeutic prompts reliably. In Week 3, therapists suggested that SARs would be perfect for tasks related to consistency and repetition but stressed that emotional tone and responsiveness would be most critical to prevent children from feeling alienated or overstimulated. These expectations were usually framed in therapeutic interests, i.e., session flow and child trust.

Lastly, SLP attitudes shifted from speculative curiosity to conditional enthusiasm, contingent on what they knew of client needs and evidence-based practice. Their hope was not based on innovation but on the potential for SARs to support their clinical skills without overwhelming the human relational aspects of therapy.

### 5.2 RQ2: How does an SLP encourage engagement through nonverbal communication?

Clinicians framed SARs as adjunctive tools that could be integrated easily into organized therapy schedules. Themes in Week 2 focused on delegation, with SLPs envisioning SARs augmenting warm-ups, transitions, or supported practice but not substituting for therapist-led instruction. Participants pictured SARs providing visual schedules, modeling social behavior, and reinforcing turn-taking in group therapy. Participants also emphasized that expression should both be an exaggerated and neutral strategy dependent on the client.

In Weeks 3 and 4, therapists outlined more tangible integration ideas, such as using SARs to practice pragmatic language with children who enjoy low-stakes peer simulations. Some therapists indicated that SARs might be models of social behavior—-providing scaffolded feedback in a consistent, judgment-free voice. Others recommended using SARs within behavioral routines to cue regulation or introduce an activity with predictable prompts. Nonetheless, clinicians stressed that SARs must be flexible with regard to a number of therapy objectives, session formats, and client groups.

Successful integration would depend on SARs augmenting—not replacing—therapist roles. SLPs outlined a co-facilitation model, where SARs offer assistance with maintaining attention, delivering consistent cues, and supporting affect regulation, under the therapist’s guidance. The proposed application was modular, whereby SARs would conduct highly structured or repetitive tasks while the therapist might attend to interpretation, personalizing, and relational engagement.

This vision instructs SAR designers to develop robots that become transparently integrated into the clinician’s workflow. It also implies the necessity of training materials and protocols that cue SLPs to integrate SARs gradually, as comfort and session demands dictate.

### 5.3 RQ3: How do the experiences of SLPs influence their opinions and willingness to use a SAR during interventions?

SLPs unanimously asserted that SARs need to be responsive, expressive, and attuned to children’s social cues. Week 3’s discussion demonstrated very well how important facial expression and emotion modeling are in speech therapy. Therapists referred to the significance of dynamic nonverbal feedback—eye gaze, gestures, and affective prosody—to establish rapport and scaffold children’s learning. SARs that cannot sense or reproduce these interactions can forfeit their therapeutic efficacy, particularly for clients with social-communication disorder.

Clinicians also referred to requirements for flexibility and customization. Successful SARs, they maintained, need to permit therapists to customize interactions—modulating cue timing, prompt content, and pacing as required. Participants dreaded over-reliance or inflexible automation, warning that non-adaptable SARs might disrupt session flow. Or clash with therapeutic objectives. Although participants appreciated elements of behavioral tracking and articulation analysis, they emphasized that such features must be under clinician control.

Contextual limitations also shaped clinician attitudes. Inadequate funding, inconsistent technical support, and vague training routes were common challenges. Phased, incremental roll-outs with a beginning focus on straightforward tasks and growth only as systems proved themselves reliable and of value in real-world practice settings were preferred by most.

Above all, participants wanted to be involved in the development process itself. Co-designing with SLPs was perceived as essential to help SAR behavior and presentation align with the realities of therapy—especially timing, expressiveness, and responsiveness to the needs of individual clients.

For SAR designers, these results highlight the importance of clinician-controlled systems. Elements need to be programmable, flexible, and responsive to the behavior of the child and the therapist’s strategy. Instead of enforcing rigid workflows, SARs need to provide module elements that therapists can use to apply and modify in real time.

Critically, SARs are not substitutes for therapeutic relationships. Their strength lies in augmenting structured routine and modeling, yet not replacing human understanding or emotional sensitivity. SLPs noted that SARs should be used as a supplemental tool for intervention that is easy to adapt and control from a clinician’s perspective. If SARs guide interactions or respond in an out-of-proportion manner to the child’s behavior, they compromise therapeutic gains.

### 5.4 RQ4: What is required for the acceptance and usability of a SAR agent during an intervention?

Therapists demonstrated a fundamental principle: SARs ought to be supportive aids, and not substitutes, for human-led intervention. They are useful only in circumstances that augment and not overwhelm clinician-child interaction. Robots getting in the way of emotional rapport or session flow will not succeed in practice.

To achieve wider use, SARs need to be adaptable to a wide variety of therapy objectives, client requirements, and environments. Participants proposed user-configurable performance, tunable expressiveness, and therapist-controlled interfaces as essential design elements. More significantly, effective integration will be not just a product of technical design but also compassionate implementation—gradual roll-outs, lengthy demonstrations, and clinician training are imperative.

Acceptance also hinges on how well SARs fit with institutional environments. Clinicians stressed usability factors like setup time, interface ease, and trouble shooting. Tools that disrupt session flow or demand too much intervention will not be accepted. Instead, SARs need to function smoothly and unobtrusively within current routines to augment and not encumber the therapist’s practice.

Briefly, SAR acceptance relies on contextual and emotional sensitivity, responsiveness in real time, and low-friction user experience—founded on solid clinician engagement and control.

### 5.5 RQ5: What development considerations would be most helpful based on the preferences of SLPs?

Recommendations drawn from these themes include.

1. Develop SAR interfaces that prioritize clinician control, allowing therapists to select tasks, cue timing, and reinforcement strategies.
2. Integrate visual and auditory expressiveness to support pragmatic skill-building, especially for children with autism or social communication disorders.
3. Create tiered implementation pathways, beginning with simple tasks (e.g., visual schedules, role-play modeling) before advancing to articulation feedback or data logging.
4. Provide clinicians with structured on-boarding resources and evidence-based use cases to build confidence and alignment with intervention goals.

By matching SAR capacities and clinician agendas, we can drive meaningful, long-term adoption. The thematic findings over the 4 weeks underscore not just what SLPs wish SARs would do, but how and why they would (or would not) integrate them into practice. Each week added richness to the design challenges, emphasizing that SAR incorporation is not just a question of function, but of fit—fit with therapeutic relationships, session dynamics, and client-responsive goals.

Another cross-cutting result was the growing realization of SAR potential. From initially being unknown, clinicians became progressively more at ease with where SARs would be useful, for example, in systematic peer modeling, social-emotional cue practice, or as a behavioral reward agent. This was, however, balanced with guardrails regarding the need to preserve therapeutic depth, emotional subtlety, and human connection. That the clinicians can consider high-leverage use cases while also indicating possible pitfalls, e.g., client over-reliance or distraction—attests to a nuanced mental model of what good therapy is and what technologies need to do in order to facilitate it.

Last, we embody a delicate balance between creativity and feasibility. Passion for rich, emotive, and interactive content was tempered by the realities of low budgets, limited training hours, and organizational support. This tension addresses a co-designed, staged deployment plan in which initial SAR deployments are aimed at particular low-risk use cases and are engineered with clinician feedback loops. Longitudinal studies with practicing SLPs should be continued in future research to determine the impact of SARs on clinical outcomes, workflow patterns, and child engagement over time. It is only in this circular process that SARs can be transformed from speculative tools to actual, relational partners in communication therapy.

### 5.6 Design and deployment implications

The incorporation of SARs into SLP treatment is a promising possibility, but one that also demands earnestly matching robotic technology with high-level social, affective, and therapeutic skills of human therapists.

#### 5.6.1 Grounding SAR design in therapeutic expectations

SARs also need to support dynamic pacing of real-time interaction, especially when sessions deviate from preplanned activities in accordance with child responsiveness or novel behavioral needs. SLPs reported regular in-the-moment strategy changes, tone changes, and activity structure changes—necessitating assistive technologies capable of pausing, changing, or redirecting in synchrony with them. Grounding robotic interaction protocols in these situational realities, SARs can emerge as genuine collaborators in a highly responsive and flexible therapy space. Instead of adding robotic capabilities to a design for a generic classroom setting, developers need to attune robotic behavior to the relational work of SLPs. Clinicians were clear that therapy is not a series of mechanistic prompts but a fluid, emotionally responsive interaction. This requires SARs to be designed with routines that can be calibrated to individual pacing, cue responsiveness, and emotional attunement. Designers must research therapy schedules, documentation requirements, and child behavioral patterns in an effort to incorporate robotic functions that represent therapeutic realism.

#### 5.6.2 Prioritizing social intelligence and embodied communication

The SLPs indicated that therapeutic interaction success is dependent on affective attunement and the capacity to reflect or validate an inner state of the child. To engage SARs in a meaningful way, they need to express sensitivity to fundamental emotion recognition and reflect facial affect in a manner consistent with appropriateness. SARs also need to be capable of moving smoothly from one expressive state to the next and reflect subtle variations in tone or gesture to fit context. Without these capabilities, SARs can potentially derail rapport, decrease engagement, and deliver prompts or feedback that are emotionally discordant or jarring. SLPs employ facial expressions, posture, and intonation chiefly to scaffold child responses and create emotional rapport. These social cues are requisite. SARs wishing to engage in a meaningful manner must transition from canned animations to express dynamic, context-dependent affective responses. These include synchronized blinking, gaze following, fine-grained prosody, and turn-taking gestures. These will allow SARs to serve as peer models or co-regulators in social-pragmatic skill sessions. Without these, robots risk being ignored, misunderstood, or even rejected by clients as incompetent agents.

#### 5.6.3 Customization, control, and clinician autonomy

Clinicians stressed that any session, indeed any moment in a session, can require alternative robot behaviors as a function of client energy, frustration, or changing therapeutic goals. SAR interfaces need to allow rapid toggling, session-defined presets, and instant therapist override. Including therapists in the design of such interfaces not only guarantees usability but also generates the trust and ownership necessary for extended use. This encompasses allowing therapists to choose tasks, adjust cue intervals, terminate interactions, or change modes during a session. Therapists stated a potential need for SARs to maximize therapeutic methods that they have already identified. The user interface must therefore be intuitive to use, customizable to individual clients, and sensitive to the therapist’s objectives, as well as the child’s attention. These choices also demonstrate concerns for responsibility and ethical alignment, since therapists are liable for interventions done under their own names.

#### 5.6.4 Institutional and contextual fit

These results also create the imperative to design SARs with resource variation in mind. The creators must build solutions that scale and behave predictably in a variety of technical environments—whether in well-resourced clinics or poorly resourced public school settings. Simplified deployment procedures, minimal maintenance care, and offline functionality would go far towards greater adoption viability. Additionally, cross-disciplinary collaboration with administrators, IT personnel, and other support personnel will need to provide long-term operational feasibility. SLPs reported logistical and administrative constraints—caseload size, lack of setup time, and lack of school leadership support—that restrict the viability of implementing new technology. Therefore, SAR implementation needs to take into account support infrastructure: available training, technical support, and peer mentoring systems are crucial. Roll-out plans need to be context-sensitive as well, with the possibility of pilot testing SARs in lower-stakes environments like social groups or articulation drills prior to more material integration. The school administrators and clinic managers also need to be involved early to establish resource alignment and sustainability.

#### 5.6.5 Re-framing SARs as therapeutic partners

Since SARs are meant to augment, not replace, therapist-child interaction, they can be used as potent relational tools. Robots, for example, can provide repetitive positive reinforcement, model a behavior children imitate with the therapist, or provide a buffer for tough transitions. Perhaps most importantly, these configurations need to keep the therapist as emotional anchor and intervention director. The therapist dictates tone and therapeutic course; the SAR reinforces it with consistency, novelty, and responsiveness—without being the center of the session. In this context, SARs can be motivational friends, rehearsal partners, or regular feedback givers that reinforce between- or within-session therapeutic tasks. This role is one of re-calibrating expectations: SARs are most effective when they follow instead of leading, reinforce instead of evaluating, and scaffold instead of correcting. By conceptualizing SARs as partners in interaction—instead of replacements—institutions and developers can create systems that maintain the integrity of therapy while responding to the growing demand for novel, child-centered support.

The implications of this study are vital for the future development, implementation, and research of SAR utilization in speech-language pathology. First and foremost, the findings of this study highlight the necessity of clinician participation from the earliest stages of SAR development. Collaborative, clinician-driven development processes will be more inclined to produce robotic devices that resonate with therapeutic values, practices, and expectations. This collaborative spirit should not only influence hardware and software features but also pedagogical approaches SARs embody.

Second, the focus on nonverbal communication, emotional attunement, and moment-to-moment responsiveness identifies a central design deficit that SAR developers need to overcome. To succeed as believable co-facilitators of speech therapy, SARs need to consider more than basic prompts and animations and instead convey socially attuned behaviors that would appeal to children’s developmental agendas. This calls for face-speech synchronization, modeling turn-taking, and mimicking affective feedback—a tall order, yet an essential hurdle for the therapeutic relationship.

Third, SLPs’ conditional interest implies that adoption will hinge as much on implementation strategy as technological innovation. Training modules, school district buy-in, and demonstration opportunities will prove essential to turning curiosity into action. Institutions would be well-advised to invest in staged pilot programs in which SLPs can test-drive SARs under low-stakes conditions, solicit feedback, and iteratively develop use cases.

Lastly, the larger implication is in the reconfiguration of the place of assistive technologies in human services. More than being seen as substitutes or panaceas, SARs can flourish as adaptive, context-aware tools that augment therapist effort and extend client engagement. In this sense, SARs are not just technological advancements but relational catalysts that augment, not replace, the human element of therapy.

### 5.7 Limitations

Although our asynchronous remote format facilitated ongoing and flexible participation, it also constrained the possibility of real-time clarification or co-design iteration. Participants were largely recruited from U.S.-based clinical networks, which could impact generalizability. Moreover, the study prioritized therapist views without observing robot use in practice, which might have deepened or nuanced participant feedback. We further acknowledge that some participant quotations were concise or generalized, requiring interpretation when matching responses to sub-themes. Future studies can consider triangulating asynchronous responses with live workshops, prototype testing, or session observation.

### 5.8 Future work

Given these findings, the subsequent step is to create functional SAR prototypes in partnership with SLPs who work within varied service contexts. These prototypes need to be iteratively piloted in real therapy settings to facilitate real-time responsiveness, emotional resonance, and flexibility in features that can be personalized to meet individual clients. Research needs to investigate the differential effect of SARs with various cohorts of clients—namely individuals with ASD, selective mutism, or pragmatic language delay—to investigate how SAR functions are likely to differ by diagnosis, age, and session type.

A second direction for future research is long-term SAR adoption strategies. Most existing work addresses short-term interactions, which are prone to be affected strongly by novelty; future work must follow how SARs are maintained in treatment plans over the course of months or academic years. This involves investigating barriers to long-term use like breakdowns, changing institutional priorities, or user burn-out. Trajectories will be critical to informing scalable deployment procedures and maintenance protocols.

Finally, novel studies must be created to determine measures that assess SAR contributions outside of engagement, such as child learning outcomes, therapist workload, and parent satisfaction. Mixed-methods studies integrating observational data, standardized measures, and stakeholder interviews can help to determine the complete ecosystem effect of SAR integration.

A requirement also exists for SAR development platforms to enable modular, scalable SAR designs. SLP requirements should be variable, a plug-and-play system where software modules and expression systems can be adapted to therapy targets would significantly improve SAR utility and uptake. Open-source platforms or community-shared design libraries could propel community-based development in accordance with clinical evidence.

Finally, future research will need to address equity and policy concerns. SAR availability will, by necessity, be district and population-specific. Studies should investigate models of equitable distribution, cost-sharing, and co-creation with communities. As SARs are increasingly integrated into clinical care, vigilance that their development continues in forms that are reflective of the diversity and complexity of clinical reality will be necessary to responsible and effective innovation.

### 5.9 Conclusion

This paper presents a grounded, clinician-focused appraisal for the ways in which socially assistive robots could augment speech-language pathology in practice. Through prolonged engagement with SLPs across 4 weeks, we drew out not just the promise of SARs but the nuanced, contextually rich decision-making that would need to inform their development. Instead of foregrounding technological innovation, SLPs foregrounded therapeutic fit—querying how a robot could support routines, honor child affect, and augment therapeutic practice.

These results highlight the value of a co-design process in which clinician intuition not only informs SAR behavior but also system design. Throughout all of the themes, the overarching observation is that SARs need to be integrated with the therapist’s sensitivity, intuition, and objectives. Facial expression, adaptive pacing, and role-based delegation are not added features—they represent the basis of usable, meaningful robotic integration.

As SLPs walk a tightrope between structure and responsiveness, SARs also need to demonstrate flexibility within consistent scaffolds. Instead of task substitution, if SARs are to be embraced as therapeutic collaborators, they need to subtly cultivate therapeutic values: development, intentionality, and empathy. By design, training, and deployment that focuses on highlighting interaction models in therapy, SARs can decrease workloads, enhance participation, and engender richer communication.

Effective SAR integration into SLP practice demands ongoing conversation, iterative prototyping, and ongoing collaboration among researchers, therapists, and developers. It is only by respecting the richness of therapeutic relationships that we can achieve SARs that are not merely endured, but embraced in the communicative heart of care.

## Data Availability

The original contributions presented in the study are included in the article/supplementary material, further inquiries can be directed to the corresponding author.

## Footnotes

1
https://www.asha.org/

2
https://www.theraplatform.com/

3 This study was supervised by University of Nevada, Reno’s Research Integrity Office, IRBNet ID: 2215653.

4
https://www.ultimateslp.com/
